# Thermographic Patterns of the Upper and Lower Limbs: Baseline Data

**DOI:** 10.1155/2015/831369

**Published:** 2015-01-13

**Authors:** Alfred Gatt, Cynthia Formosa, Kevin Cassar, Kenneth P. Camilleri, Clifford De Raffaele, Anabelle Mizzi, Carl Azzopardi, Stephen Mizzi, Owen Falzon, Stefania Cristina, Nachiappan Chockalingam

**Affiliations:** ^1^Faculty of Health Sciences, University of Malta, Msida MSD 2080, Malta; ^2^Faculty of Health Sciences, Staffordshire University, Stoke-on-Trent ST4 2DF, UK; ^3^Faculty of Medicine and Surgery, University of Malta, Msida MSD 2080, Malta; ^4^Centre for Biomedical Cybernetics, University of Malta, Msida MSD 2080, Malta

## Abstract

*Objectives*. To collect normative baseline data and identify any significant differences between hand and foot thermographic distribution patterns in a healthy adult population. *Design*. A single-centre, randomized, prospective study. *Methods*. Thermographic data was acquired using a FLIR camera for the data acquisition of both plantar and dorsal aspects of the feet, volar aspects of the hands, and anterior aspects of the lower limbs under controlled climate conditions. *Results*. There is general symmetry in skin temperature between the same regions in contralateral limbs, in terms of both magnitude and pattern. There was also minimal intersubject temperature variation with a consistent temperature pattern in toes and fingers. The thumb is the warmest digit with the temperature falling gradually between the 2nd and the 5th fingers. The big toe and the 5th toe are the warmest digits with the 2nd to the 4th toes being cooler. *Conclusion*. Measurement of skin temperature of the limbs using a thermal camera is feasible and reproducible. Temperature patterns in fingers and toes are consistent with similar temperatures in contralateral limbs in healthy subjects. This study provides the basis for further research to assess the clinical usefulness of thermography in the diagnosis of vascular insufficiency.

## 1. Introduction

Physiological processes produce heat. Since body temperature is an indicator of normal or abnormal function, altered body temperature is a natural indicator of disease [1]. Several studies report that an increase >2.2°C (4°F) may require further investigation [2]. The core temperature of the human body is normally maintained at 37°C. Changes in temperature at the peripheries can occur as a result of environmental changes with the body's response to this being peripheral vasoconstriction or vasodilatation to control core temperature, together with other physiological mechanisms such as sweating, to encourage or prevent heat loss.

This normal thermoregulatory response may be altered by certain pathologies such as peripheral arterial disease which can cause changes in peripheral temperatures, resulting in cooler extremities. Furthermore, in certain inflammatory conditions, such as local infection and gout, the peripheral temperature will increase as a result of local vasodilatation mediated by cytokines elicited by the inflammatory process. In autonomic neuropathy, these normal thermoregulatory processes are impaired, resulting in altered peripheral temperatures due to impaired neurovascular function [3].

Areas of increased temperature in the foot are predictive of the development of ulceration [4]. Presently, the common clinical practice of temperature assessment involves manual palpation of the foot. However, the temperature changes are typically too small to be reliably determined using this technique. This practice does not rely on quantitative and repeatable methods and is unlikely to be reliable in assessing multiple temperature readings in various parts of the foot. A reduction in peripheral perfusion to one or more lower limbs may lead to a subtle reduction in temperature compared to a normally perfused upper limb, which will not be detectable through manual palpation, as opposed to thermographic techniques which can detect temperature variation with a much higher resolution. Using thermographic techniques, which are noninvasive, skin temperature may be measured without direct contact, thus providing quantification of cutaneous heat radiation [5].

Thermography is an imaging technique used to detect infrared radiation emitted from the surface of an object. Since the radiated infrared energy emitted is related to the object's temperature, with the use of thermography it is possible to determine the temperature and temperature variations of this object of interest. The technology is widely employed for a range of applications within both commercial and industrial environments. In the medical field thermography has been used in oncology, dentistry, urology, and dermatology [1].

In this work, the primary interest is in the application of thermography for the physiological imaging of peripheral circulatory function of hands and feet and for its use as a clinical tool for the diagnosis and prognosis of peripheral vascular disease and neurological disorders [6]. A thermal imaging system makes it possible to obtain an accurate measure of skin temperature in a noncontact manner through cutaneous heat radiation. Every material has an associated emissivity value which represents the material's effectiveness in emitting thermal radiation. This value can range from 0 to 1, where 1 refers to the emissivity of an ideal black body which radiates the highest amount of thermal energy that can be emitted at a specific temperature. Materials with an emissivity close to 1 are particularly adequate for thermographic imaging because the radiated heat is closely related to the actual surface temperature. With an emissivity value of approximately 0.98, human skin is thus particularly suitable for temperature measurement using thermography [1, 5]. In fact the use of noncontact infrared thermography can provide a more accurate reading than contact methods [7]. The interested reader is referred to Lahiri et al. [1] and Ring et al. [8] for further literature which discusses the physical concepts behind thermographic imaging and a review of its application to medical problems.

The use of thermography in the context of the high risk foot has been reported in prevention strategies and in predicting skin breakdown, impending ulceration [4, 9], and predicting optimum level for amputation. Objective measurement of skin temperature has been shown to predict amputation healing with an accuracy of 80 to 90% [10]. Beyond these areas however the use of thermography is limited and has not been adopted widely in the clinical setting despite early promise of its potential clinical application [11].

One of the main reasons for the limited use of thermography is the present prohibitive expense of high-end thermography equipment. However, beyond cost, use of thermography in the context of the high risk foot, namely, the diabetic foot, has been problematic because of the lack of accurate reporting of thermographic findings both in healthy subjects and in diabetic patients. Thermographic data from diabetic feet has tended to be interpreted in isolation from the rest of the body apart from some studies where data from the contralateral limb has been used.

Currently the ankle-brachial pressure index (ABPI) is one of the most common physiological tests for perfusion of the lower limbs. The patient's own upper limbs serve as the denominator in the ABPIs. This is necessary since the absolute systolic pressure is highly variable between individuals and in the same individual at different times of the day and in different circumstances. As a result isolated measurement of systolic pressure at the ankle would yield unreliable and hugely variable results from the same individual. Comparing the ankle systolic pressure to the brachial pressure however eliminates the problem of variability in absolute pressures and yields a reliable and cheap tool for assessment of perfusion. The same principle could prove useful if thermographic patterns in the lower limbs are compared to those in the upper limbs which are relatively spared of arterial disease even in the context of severe peripheral arterial disease. Before this is possible, however, it was necessary to investigate thermographic data and patterns in the upper limbs in healthy controls. This was the basis for including assessment of thermographic patterns in the upper limbs in this study.

The major advantage provided by the use of thermography over currently employed clinical vascular measurement techniques of ABPIs, toe pressures and spectral waveforms, is that this technique involves no direct contact with the skin, thus reducing risks of infection, and it is relatively quick. It also has the potential of assessing the effects of both neuropathy and arteriopathy on the foot in one test. The main advantage would be in the completeness of the assessment. Current tools such as ABPIs only provide information about perfusion to the level of the ankle and are limited in the case of diabetes, chronic kidney disease, and old age where calcified arteries may yield artifactually elevated results, implying unreliability of the ABPI test itself [12]. Toe brachial pressure indices are only taken from the big toe or occasionally the second toe and do not provide information about perfusion to the remaining part of the foot which may be very different as newer concepts of angiosomes have shown.

Thermography has the potential of providing an assessment of the whole foot by looking at different parts of the foot separately and comparing them to the contralateral foot and more proximal parts of the limb as well as the upper limb. It is also repeatable and noninvasive allowing the tool to follow patients over time and assess the effects of intervention such as surgical or endovascular revascularisation, as well as the effect of antibiotic treatment in diabetic foot infections.

One of the disadvantages of infrared imaging is that it can provide physiological information; however, it cannot define aetiologies and local anatomy [13].

Although various studies [14–16] have attempted to quantify skin temperature as a measure of pathological vascular changes in the diabetic foot, Sun et al. [17] report limited usefulness of this technique because of poor measurement methodology and procedures. On the other hand, various authors concluded that thermography could be regarded as an emergent potential diagnostic tool [18–20] that assesses circulatory status both in routine foot assessment and before and after vascular interventions.

Previous work, albeit limited, has been carried out to establish some normative baselines in terms of temperature measurements of different body areas using thermography [21–24]. These studies have assessed the mean temperatures at different sites and, in certain cases, evaluated thermal symmetry between contralateral sides of the same body parts.

In view of the paucity of evidence in this field, the objectives of this research were to determine the normative heat pattern distribution in feet and hands in a population of healthy adults, to identify any possible differences in heat pattern distribution between both the contralateral and ipsilateral feet and hands, from which a clinical protocol for the acquisition of reliable thermographic and visual data for the feet, legs, and palms of a recruited sample could be developed.

## 2. Materials and Methods

Ethical approval was sought and granted by the University of Malta Ethics Research Committee. All participants provided informed consent to participate in this study. The reported investigations were carried out in accordance with the Declaration of Helsinki as revised in 2000 [25].

This single-centre, randomized, prospective study was conducted on 63 healthy adults without a history of significant medical, surgical, vascular, or neurological disease. Those with an ABPI <1 or >1.3 were excluded, since this is the most widely used test for diagnosing peripheral arterial disease (PAD) [12], having been reported as being 100% specific in identifying healthy individuals [26]. Participants with a history of smoking or alcohol abuse were also excluded. All included participants demonstrated normal triphasic waveforms as reported by a hand-held Doppler.

Demographic data was recorded for each participant, together with anthropometric measurements which included weight and height. The testing modalities and examination methods were carried out by the same two clinicians with over 10 years of experience in the field to ensure uniformity. Testing was performed in the same examination room at a controlled mean temperature of 22.6°C (SD 0.39) (at 55%–60% relative humidity), which was monitored using a calibrated thermometer.

### 2.1. Testing Protocol

Measurement of ABPI was performed using a portable hand-held Doppler and blood pressure cuffs. Additionally, quantitative pedal waveform analysis of the dorsalis pedis and posterior tibial artery was obtained from all recruited subjects utilizing the continuous wave Doppler. These waveforms and the measurement of the resting ABPI were obtained using the Huntleigh Dopplex Assist Vascular Package (Cardiff, UK) as the principal study tool. The 8 MHz probe was held steadily on the anatomical artery location at an angle between 45 and 60 degrees against the flow of arterial blood. Interpretation of arterial pressure waveforms results was based on standards from the literature [18]. Waveforms were classified as triphasic, biphasic, monophasic discontinuous, and monophasic continuous. Triphasic waveforms were considered normal, whereas the biphasic and monophasic discontinuous and monophasic continuous waveforms were interpreted as abnormal and indicative of PAD. As triphasic waveforms were essential criteria for inclusion, participants with other waveforms were excluded. Measurements were carried out after a 5-minute rest in a supine position with the upper body as flat as possible. Patients were also asked to undo all tight clothing around the waist and the arm. A blood pressure cuff was applied to the arm (to measure the brachial systolic pressure) and the ankle (to measure the dorsalis pedis and posterior tibial pressures) to determine the ankle pressure. The cuff was inflated to occlude the arterial pressure. The systolic pressure was obtained by listening to and noting the pressure on the manometer. The higher values of the brachial and the ankle pressures were used to calculate the ABPI. Values were interpreted according to the criteria proposed by the American Heart Association and the American Diabetes Association [19].

All participants underwent a standard neurological examination which included a 10-gram Semmes Weinstein monofilament test performed on both feet to ensure the absence of peripheral sensory neuropathy [27]. The plantar aspect of the hallux and the 3rd digit, together with the 1st, 3rd, and 5th metatarsal heads, were used for testing. With the eyes closed, the participant related to the investigator whether he or she could feel the monofilament. The ability to feel all the 5 points tested was considered to be indicative of normal neural function and was a prerequisite for inclusion into the study.

For the purpose of data analysis, each foot was scored separately. All data was recorded on a spreadsheet designed in Microsoft Excel to group together the information required for interpretation of the results.

### 2.2. Thermographic Data Acquisition

Prior to thermographic data acquisition, participants were allowed to acclimatize to their ambient temperature for a period of 20 minutes, in concordance with standard recommendations followed in the literature [1, 4, 18, 21]. During this period of time, clinical examinations took place, while the participants were also monitored using the thermography camera to confirm that the acclimatization process was taking place. Participants were also asked to avoid using deodorants, antiperspirants, or other cosmetics that could affect the acquired thermographic pattern.

**Figure 1:**
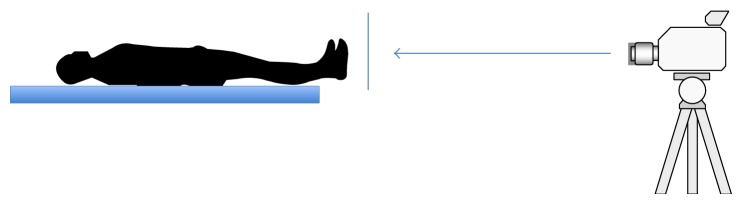
A diagram showing data capture, with the participant lying supine while a thermographic image of the plantar surface was taken. The angle of measurement was kept approximately perpendicular to the plane of acquisition.

**Figure 2:**
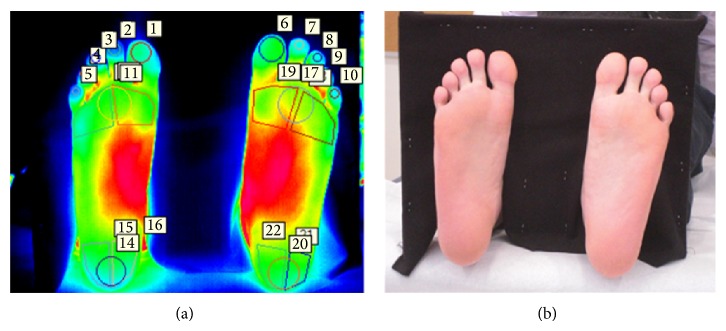
Participants were asked to keep their feet momentarily in slight dorsiflexion and pointing vertically upwards, while a thermal image (a) and a visual image (b) were acquired. Measurement regions were then annotated on the thermal image (a) for further analysis.

Following the said acclimatization period, images were taken with participants lying down, barefoot, on a couch in the supine position. A thermal camera (FLIR Model SC 7000; FLIR Systems, Inc., Oregon, USA) and a digital camera (CASIO Exilim) were mounted on separate tripods at a distance of 1.5 metres away from the end of the couch. The cameras were kept as close to each other as possible in order to retain a similar viewing angle. The thermal camera was kept perpendicular to the plane of acquisition, as shown in Figure 1. Literature states that during thermographic imaging angles of measurement of up to 20 degrees have a negligible effect on the acquired temperatures [28]. Participants were instructed to keep their feet momentarily in slight dorsiflexion and pointing vertically upwards, while a thermal image and a visual image of the plantar aspect of the foot were taken with both cameras, respectively, as shown in Figures 2(a) and 2(b). A uniform backdrop was placed on the dorsal side of the feet in order to generate a significant difference in contrast between feet and background in the thermal image.

**Figure 3:**
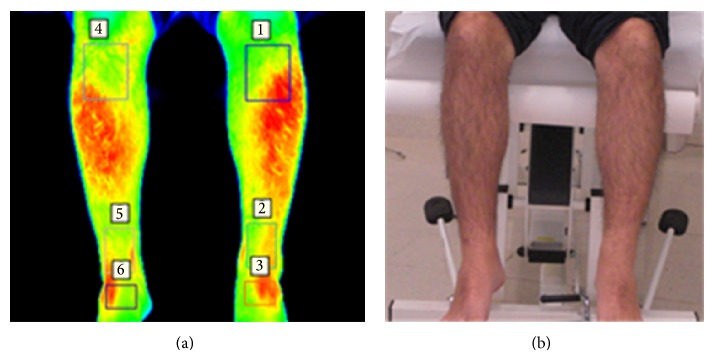
Participants were asked to hang their legs over the edge of the couch, with their feet held momentarily in slight plantarflexion, while a thermal image (a) and a visual image (b) were acquired. Measurement regions were then annotated on the thermal image (a) for further analysis.

A second set of images was taken with participants in a sitting position with their legs hanging over the edge of the couch, with their clothing rolled up to the knees, and with their feet held momentarily in slight plantarflexion. Both a thermal and a visual image of the dorsal aspect of the foot and the anterior aspect of the shins were taken with both cameras against the backdrop, as shown in Figures 3(a) and 3(b).

**Figure 4:**
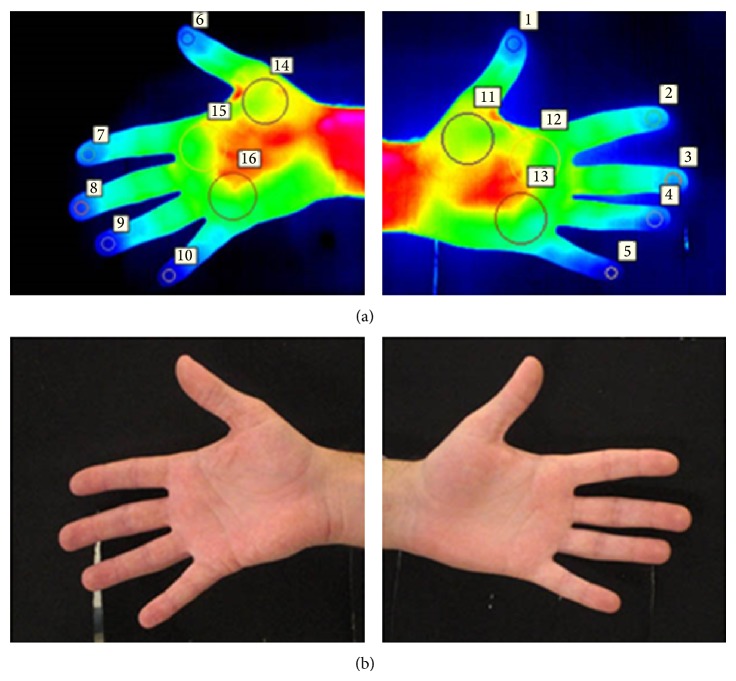
Participants were asked to spread the palms and fingers of each hand in front of the cameras one at a time, while a thermal image (a) and a visual image (b) were acquired. Measurement regions were then annotated on the thermal images (a) for further analysis.

A third and final set of images of the volar surface of both hands was captured, with the participants standing up with their palms and fingers spread in front of the cameras one at a time, as shown in Figures 4(a) and 4(b). The palmar aspect of the hand was chosen because it is comparable to the plantar aspect of the foot in anatomical terms.

### 2.3. Thermographic Data Measurement

Thermographic temperature measurements of the sites of interest of each participant were extracted from predefined measurement areas using the thermographic software provided by the manufacturer (FLIR Altair). These measurement regions were annotated using a template shown in Figures 2(a), 3(a), and 4(a). The mean thermographic temperatures (*θ*_*p*_^mean^) were tabulated for each region for each participant *p*.

## 3. Results

Out of a total of 67 prospective participants, 63 healthy adult participants, 24 males and 39 females with a mean age of 36 years (SD 12.24), were included in the study. Four individuals were excluded during screening due to elevated ABPI readings or because they were on medication. Mean weight for the study sample was 70.5 kg (SD 14) and mean height was 164.5 cm (SD 9.7).

For each individual participant in the study, the tabulated temperatures within each region, depicted in Figures 2(a), 3(a), and 4(a), were initially analysed separately so as to quantify the thermal variation and distribution occurring inside each segmented area.

**Figure 5:**
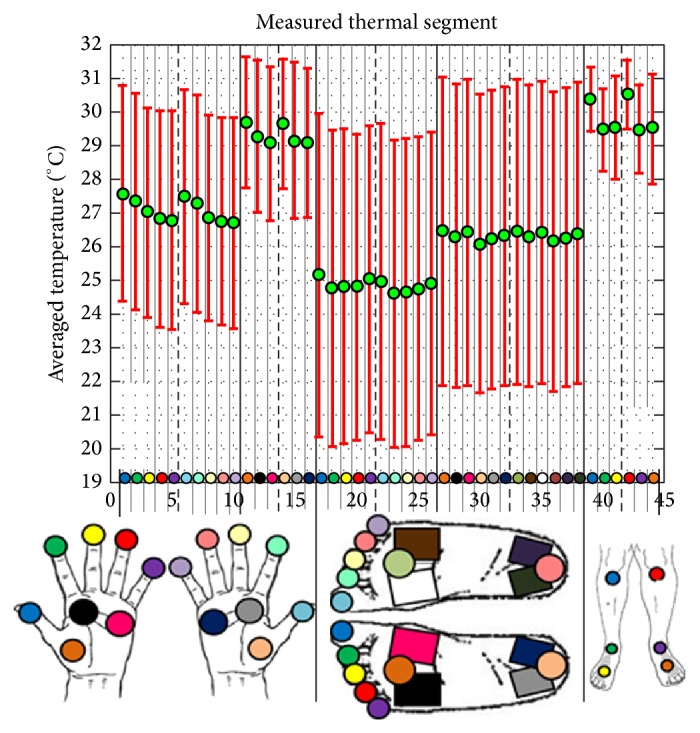
The mean thermographic temperature of each region (green dot) and the standard deviation across subjects for each region (red bars).

The mean and standard deviation across subjects for each region are shown in Figure 5 where the green dot represents the mean thermographic temperature and the red bars represent the standard deviation across subjects for each region.

In Figure 5, a number of observations may be noted—the first of which is the general symmetry in terms of mean temperatures for both sides of the participants. For extremities such as toes and fingers, or core areas such as the volar and plantar surfaces, the mean temperatures of both left and right sides of participants are considerably similar—in terms of both magnitude and pattern. The mean absolute differences between corresponding anatomical sites are noted to be statistically smaller than 0.89°C (*P* < 0.05). Another observation that may be generalized across hands, feet, and shins is that the mean temperatures of extremities such as fingers, toes, or dorsal surfaces of the feet are lower in relation to their “core” areas, namely, the volar and plantar surfaces, and shins, respectively. For both hands, the temperature difference between volar surfaces and fingertips was found to be statistically larger than 1.44°C (*P* < 0.05). For the feet, the temperature difference between plantar surface and toes was found to be statistically larger than 0.84°C (*P* < 0.05). Finally, for the legs, the temperature difference between the shins and feet was found to be statistically larger than 0.61°C (*P* < 0.05).

Figure 5 also reveals patterns in temperature variation across fingers and toes. When considering the hands, a monotonic decrease in temperature from the thumb to the fifth digit may be noted. When considering feet, the hallux and fifth digit are observed to have elevated temperatures in relation to the second, third, and fourth digits.

**Figure 6:**
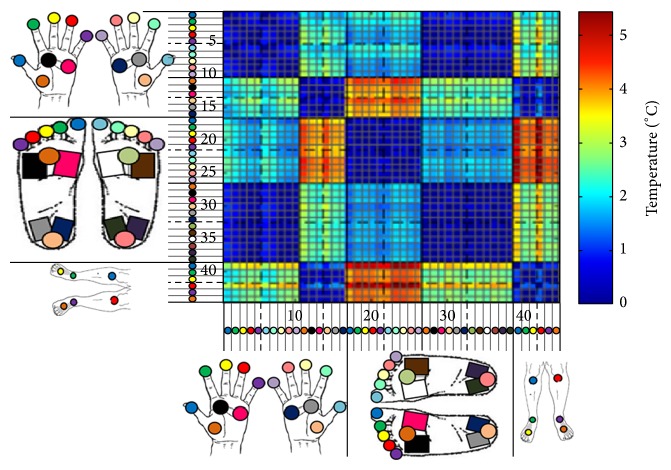
Matrix of the participant-averaged thermographic temperature mean differences between each region-pair.

**Figure 7:**
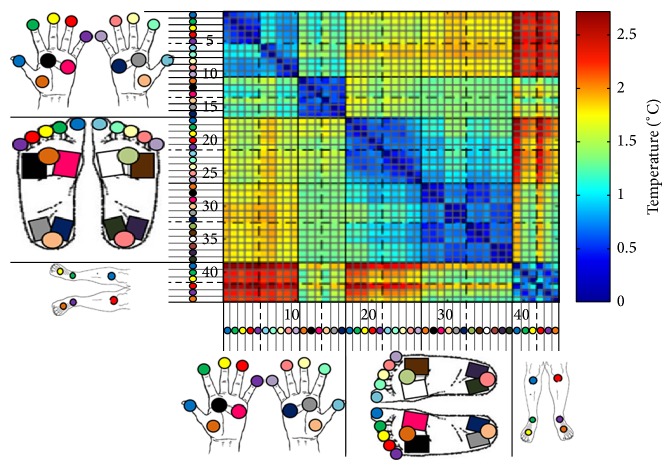
Matrix of the standard deviations of the participant-averaged thermographic temperature mean differences between each region-pair.

An investigative analysis was also performed on the difference in temperature between each region. This was done by calculating the average difference between the mean thermographic temperature of each region and that of other regions within the same participant. This was repeated for each participant to obtain the participant-averaged temperature difference between each region-pair. A colour-coded matrix was generated for visual analysis, illustrated in Figures 6 and 7, which depict the average absolute temperature difference of each region-pair and its standard deviation, respectively.

### 3.1. Interpretation of Results

The “block-like” pattern evident in the thermographic temperature difference matrix of Figure 5 shows that the thermal differences between regions inside a unique anatomical area (specifically the fingers, volar, toes, plantar, and shin areas) are very minimal when compared to the difference exhibited between regions from different anatomical areas. This categorisation into separate areas is further corroborated by the fact that the diagonal sections of Figure 5, representing the temperature similarity between neighbouring regions of the same anatomical area, have a mean temperature difference of less than 0.86°C. This is statistically consistent across participants as depicted by the very low standard deviation shown on the diagonal of Figure 6. This result enables simplification of the interpretation of the thermographic temperature difference matrix by considering similar regions as elements of disjoint sets representing unique anatomical segments which can then be more significantly compared for temperature differences between them as shown in Table 1.

It can be deduced from this data that the average temperature difference between plantar regions and fingers varies only by a maximum of 0.94°C within an individual patient with a standard deviation of 2°C. A similar temperature difference is also observed between the volar and the shins regions with a temperature difference ranging only up to 1.2°C (SD 1.6°C).

Conversely, anatomical segments which exhibited large temperature differences between them, illustrated in red regions in Figure 5, include the toes-volars segment-pair and the toes-shins segment-pair. For the former case, regions between both anatomical segments showed a thermographic thermal difference between 4.17°C and 5.12°C with a standard deviation of 1.76°C. The thermal difference resulting between measured regions on the toes with respect to regions on the shins was also quite substantial, with a minimum of 4.1°C and a standard deviation between 1.9°C and 2.9°C on the dorsal and knee regions, respectively.

## 4. Discussion

This study has shown that measurement of skin temperature in both lower and upper limbs is feasible and reproducible. It has also confirmed previous data that there is minimal difference in temperature between the same regions in contralateral limbs. The intersubject variability in temperature in a particular area in the same ambient conditions is also shown to be minimal in healthy subjects. In this study, the importance of comparing fingers to volar surfaces of hands and toes to the plantar surfaces of the feet is that in disease conditions the heat pattern in the fingers or toes may be altered as discussed previously. Knowing the normal relationship of temperatures in the fingers relating to the palm and toes to the plantar aspect of the foot in normal controls may prove useful when assessing patients with pathology.

This study has also revealed new and important information regarding the patterns of temperature both in the hands and in the feet. It has shown that, in the upper limb, the thumb is consistently warmer than the other fingers with a progressive decrease in temperature from the 2nd to the 5th fingers. One possible explanation for this could be that the thumb is the shortest of all the fingers and therefore is closer to the body core compared to the other fingers. The pattern in the foot is different with the hallux being the warmest followed by the 5th toe, while the 2nd to 4th toes have a lower temperature. In this regard the possible reason for this is that the dorsal metatarsal arteries to the big toe are the continuation of the arteria dorsalis pedis and those to the little toe the continuation of the lateral tarsal artery. The dorsal metatarsal arteries to the remaining three toes arise from the arcuate artery which is normally a smaller artery and is formed by a branch of the lateral tarsal artery and the dorsalis pedis artery. On the plantar aspect the lateral plantar artery divides into two, one branch continuing into the 5th toe, while the other then forms the plantar arch which supplies all the middle three toes. The hallux is supplied by the superficial branch of the medial plantar artery besides a branch from the plantar arch itself. This implies that the best perfused toes are the 1st and the 5th which explains the higher temperatures in the 1st and 5th toes. These patterns were observed in all subjects and appear to be consistent.

Another important finding of this study is that the temperature of the shins is practically identical to the temperature in the palm of the hand and that the temperature in the sole of the foot is practically identical to the temperature in the fingers. The biggest temperature difference is observed between the toes (particularly 2nd–4th) and the shins as well as between the toes (particularly 2nd–4th) and the palms of the hands.

The consistency of the data reported in this study in both upper and lower limbs as well as the patterns of temperature in the toes and fingers provides a solid basis on which further research relating to arterial disease can proceed. Although changes in thermal patterns may be relatively small, since the thermograms yield both qualitative and quantitative data, meaningful results can be obtained if patients are properly assessed [13]. The aim of including this technique in clinical practice is not to substitute for clinical examination but to enhance it, since this technique provides clinically important data that is not attainable through other clinical modalities. With this normative data now available, it becomes possible to further proceed to develop the required protocol for this noninvasive analysis of foot and hand thermographic scanning in order to facilitate the diagnostic process related to the various vascular pathologies.

Although the upper limbs are relatively spared of arterial disease even in the context of severe peripheral arterial disease when compared with the lower limbs, it was necessary to investigate thermographic data and patterns in these limbs in healthy controls in order to provide the necessary basis for the formulation of a protocol on the use of thermography in disease. Future research will need to establish whether interpreting thermographic data from the lower limb in conjunction with data from the upper limb will provide reliable and consistent assessment of the presence of underlying disease, the type and level of disease, and the severity of that disease.

## 5. Conclusion

Measurement of skin temperature of the lower and upper limbs using a thermal camera has been shown to be feasible and reproducible. Temperature patterns across the fingers and toes are consistent with practically identical temperatures between contralateral limbs. This study has revealed specific thermographic patterns in the fingers and in the toes. Practically identical temperatures have also been identified between shins and palms as well as between soles and fingers. This study provides the basis for further research to assess the clinical usefulness of thermography in diagnosis and management of vascular related pathologies.

## Figures and Tables

**Table 1:** Mean thermal difference and standard deviation between sets of measured regions corresponding to anatomical segments.

Anatomical segment-pairs difference region	Range of the mean thermographic thermal difference (°C)
Fingers-volars	2.38–3.24
Fingers-toes	0.92–2.59

Fingers-plantars	0–0.94
Fingers-shins	2.31–4.47

Volars-toes	4.17–5.12
Volars-plantars	2.31–3.29

Volars-shins	0–1.2
Toes-plantars	1.05–2.65

Toes-shins	4.11–6.17
Plantars-shins	2.25–4.33
